# Transorbital eyebrow lacrimal keyhole approach for resection of a meningioma of the lateral wall of the cavernous sinus

**DOI:** 10.3171/2025.1.FOCVID24190

**Published:** 2025-04-01

**Authors:** Arianna Fava, Tingting Jiang, Trung Hai Vu, Rosaria Abbritti, Sébastien Froelich

**Affiliations:** 1 Department of Neurosurgery, Lariboisière Hospital, Assistance Publique–Hopitaux de Paris; and; 2 University of Paris, France

**Keywords:** transorbital, eyebrow, lacrimal keyhole, meningioma, cavernous sinus

## Abstract

The transorbital approach has emerged as a minimally invasive technique in neurosurgery. The authors present the case of a meningioma of the lateral wall of the cavernous sinus treated with a transorbital eyebrow lacrimal keyhole approach (TELKA) in a 64-year-old man with partial seizures. By removing the lip of the superolateral orbital rim, the so-called lacrimal keyhole, via an eyebrow incision, optimal microscopic and endoscopic visualization and maneuverability were achieved, avoiding dangerous ocular globe retraction. Together with the chopstick technique using angled endoscopes and malleable rotative suction, TELKA allowed a safe and effective resection minimizing soft tissue dissection, brain manipulation, and ocular retraction.

The video can be found here: https://stream.cadmore.media/r10.3171/2025.1.FOCVID24190

## Transcript

We present a case of a meningioma of the lateral wall of the cavernous sinus operated through a transorbital eyebrow lacrimal keyhole approach, named TELKA. The patient is a 64-year-old man presenting with memory deficits and recurrent episodes of speech arrest attributed to a partial seizure disorder. Preoperative CT scan and MRI revealed a left-side extra-axial lesion of the lateral wall of the cavernous sinus with perilesional edema and temporal lobe compression.

### 0:50 Rationale for Procedure.

Considering the patient’s symptoms and the temporal lobe compression, a surgery was proposed.^1,2^ The first surgical option was to perform a classic frontotemporal approach with peeling of the lateral wall of the cavernous sinus. Although this option is the most versatile and used one, some drawbacks are still present, that is, an extensive dissection of soft tissues, temporal muscle pain, aesthetic issues, brain manipulation, and retraction of the temporal lobe.

Considering the absence of encased arteries within the tumor, the patient’s baldness, and a thick eyebrow, a transorbital route was considered.^3–5^ A TELKA—transorbital eyebrow lacrimal keyhole approach—was decided. The lacrimal keyhole corresponds to the removal of the lip of the superolateral orbital rim, which covers the lacrimal gland.^6,7^

The lacrimal keyhole provides an optimal line of sight and sufficient working space, reducing ocular globe retraction.^8^ This window provides an excellent visualization and maneuverability with both microscope and endoscope.

Additional drilling of the frontal bone, greater and lesser sphenoid wings exposes the deep periosteal layer of the temporalis muscle, the temporal dura, and the orbitofrontal periosteal fold.^6,8^

### 2:05 Setup: Patient Positioning and Equipment.

The patient is positioned supine and the head secured on Mayfield holder extended and rotated 10° contralaterally. The skin incision is made into the eyebrow laterally to the supraorbital nerve, about 3 cm of length. The microscope and endoscope are prepared. Neuronavigation is set up to confirm the surgical anatomical trajectory.

### 2:27 Surgical Procedure: TELKA.

The first part of the surgical approach is conducted under microscope. The eyebrow skin and subcutaneous tissue are incised, as well as the fibers of the orbicularis muscle. The periosteum is cut along the superolateral orbital rim and dissected. The superior temporal line and the frontozygomatic suture are exposed. The periosteum is retracted and the periorbita is gently elevated from the orbital rim. The lip of the superolateral orbital rim is removed with a craniotome. The periorbita is progressively elevated and retracted by bipolar coagulation. A plastic malleable retractor is used to protect the periorbita. The bone drilling starts from the lateral wall and roof of the orbit to expose the deep periosteal layer of the temporalis muscle. This periosteal layer should be carefully preserved to keep the innervation for the muscle. The greater sphenoid wing is progressively drilled.

Continuing the drilling of the greater sphenoid wing, the temporal dura is exposed and peeled from the middle fossa floor. The last piece of bone of the lateral border of the superior orbital fissure is dissected and removed. V2 entering into foramen rotundum is exposed.

Medially the orbito-temporal periosteal fold is identified. After cutting the orbitotemporal periosteal fold, the lateral wall of cavernous sinus is peeled using an ophthalmic scalpel, Rhoton’s microdissector, and microscissors. Cranial nerves, in particular V1 and V2, are identified. Hemostasis is achieved with hemostatic matrix.

The trajectory is confirmed with the neuronavigation. Then, the temporal dura mater is opened and coagulated.

### 5:02 Surgical Procedure: Tumor Removal.

Thereafter, given the deep surgical field, tumor removal is performed using angled endoscope starting with a 30° scope and angled instruments: malleable rotative suction, coagulation, and microscissor. Careful attention is paid during coagulation and dissection of the medial aspect of the tumor.

In order to improve the maneuverability, the chopstick technique is used.^9^ The surgeon holds the camera and the malleable rotative suction with the left hand and the other working instrument with the right hand, while the second surgeon irrigates the camera and regulates the suction.

The tumor debulking continues using angled punch forceps and angled aspirators. To improve the visualization, a 45° scope is used. The tumor is dissected from the temporal cortex and vessels using the chopstick technique. At the end, a careful check of the cavity and accurate hemostasis are carried out using a 70° endoscope.

Final view of the surgical cavity appreciating the complete resection of the tumor through a satisfying opening.

### 6:33 Surgical Procedure: Closure.

The dural opening and the dead space are filled with autologous abdominal fat graft. The bone is replaced with titanium plates and screws. The periosteum, muscle, and subcutaneous layer are closed with reabsorbable monofilament suture. An intradermic skin suture is performed with a 4-0 nonreabsorbable suture.

### 7:20 Postoperative Course.

First days after surgery without severe orbital swelling. Postoperative CT scan shows a gross-total removal and no surgical complications. Three-dimensional reconstruction highlights the small craniotomy. The patient did not present any neurological deficits. The stitch is removed at day 6.

At 2 months of follow-up, the MRI shows a complete resection of the tumor, the cosmetic and functional outcomes result optimal.

## Disclosures

The authors report no conflict of interest concerning the materials or methods used in this study or the findings specified in this publication.

## Author Contributions

Primary surgeon: Froelich. Assistant surgeon: Fava, Vu. Editing and drafting the video and abstract: Fava, Jiang. Critically revising the work: Fava, Froelich. Reviewed submitted version of the work: Vu, Abbritti, Froelich. Approved the final version of the work on behalf of all authors: Fava. Supervision: Froelich.

## Supplemental Information

### Patient Informed Consent

The necessary patient informed consent was obtained in this study.
